# Supplementary material for: PanBGC: a pangenome-inspired framework for comparative analysis of biosynthetic gene clusters
Source: ISME Commun. 2025 Nov 27;5(1):ycaf225. doi: 10.1093/ismeco/ycaf225 (PMC12704434; doi:10.1093/ismeco/ycaf225)
Supplement: Supplementary_info_fig1_ycaf225 [file supplementary_info_fig1_ycaf225.pdf]

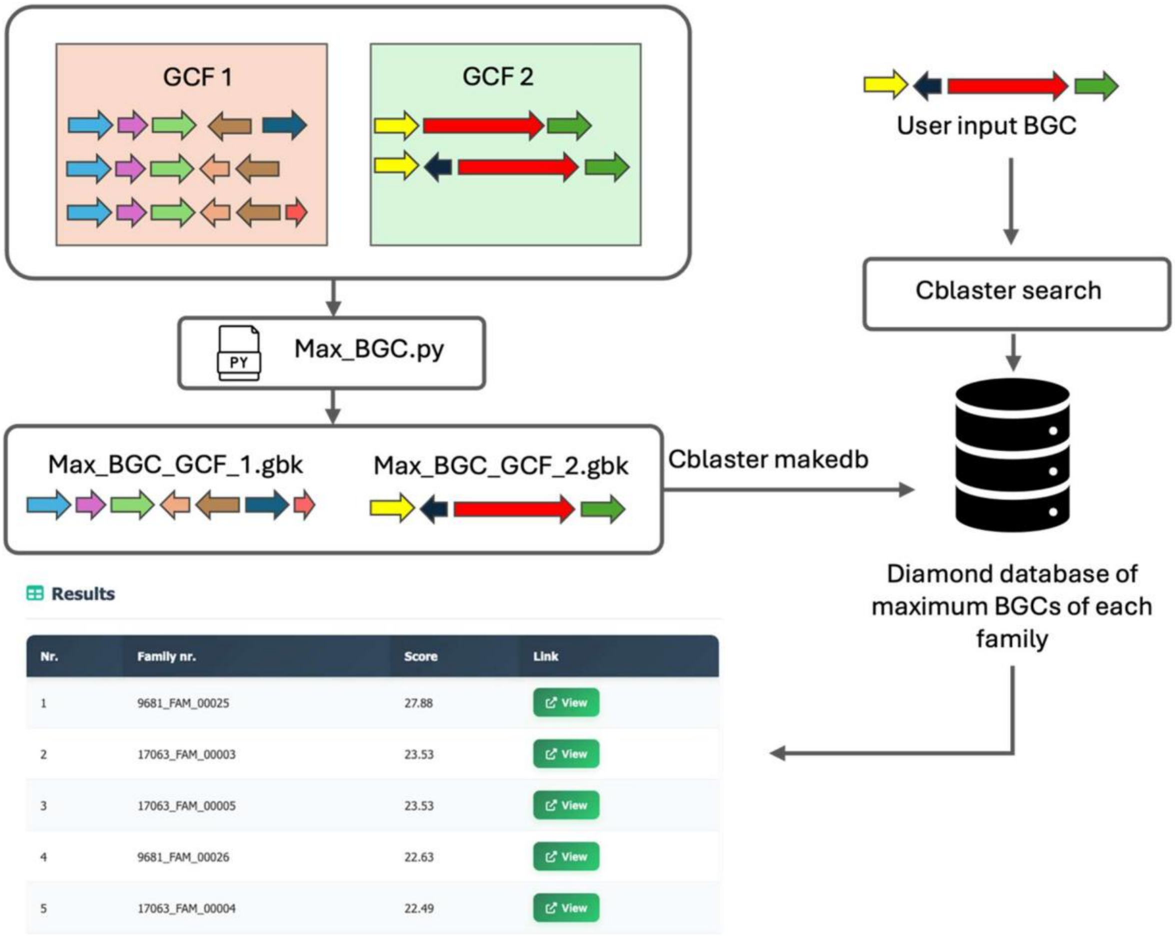

Supplementary Figure 1: Cblaster database construction pipeline and user query. Multiple GCFs results from ZOL are processed using a Python script (Max_BGC.py) to create a theoretical maximum BGC for each family. These maximum BGCs are then used to build a searchable DIAMOND database using the cblaster makedb module. A user-provided query BGC is subsequently searched against this database using cblaster search. The result identifies the best-matching family based on sequence similarity and hit coverage, which is displayed in a ranked table of candidate families.
