# Supplementary material for: PanBGC: a pangenome-inspired framework for comparative analysis of biosynthetic gene clusters
Source: ISME Commun. 2025 Nov 27;5(1):ycaf225. doi: 10.1093/ismeco/ycaf225 (PMC12704434; doi:10.1093/ismeco/ycaf225)
Supplement: Supplementary_info_fig2_ycaf225 [file supplementary_info_fig2_ycaf225.pdf]

Supplementary material

Figures

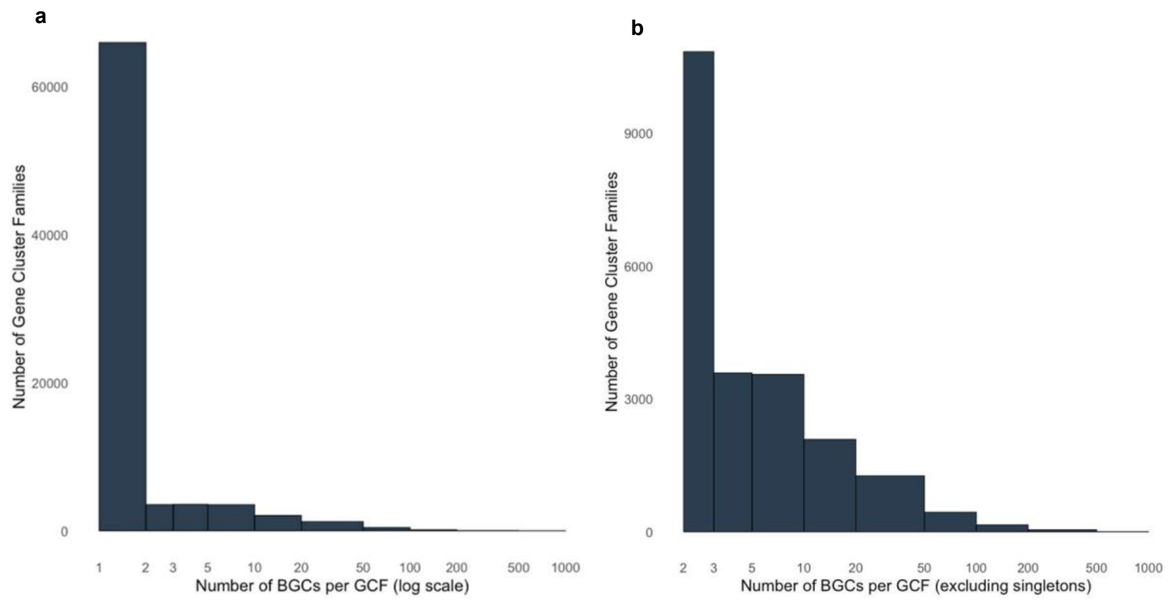

Supplementary Figure 2: Histograms showing the number of BGCs per GCF across the dataset. **a** GCF size distribution plotted on a logarithmic x-axis to highlight the long-tail structure of large families. **b** The same distribution shown on a linear scale, excluding singletons. The majority of GCFs consist of only a few BGCs, while a small subset include GCFs with hundreds of BGCs.
