# Supplementary material for: PanBGC: a pangenome-inspired framework for comparative analysis of biosynthetic gene clusters
Source: ISME Commun. 2025 Nov 27;5(1):ycaf225. doi: 10.1093/ismeco/ycaf225 (PMC12704434; doi:10.1093/ismeco/ycaf225)
Supplement: Supplementary_info_fig3_ycaf225 [file supplementary_info_fig3_ycaf225.pdf]

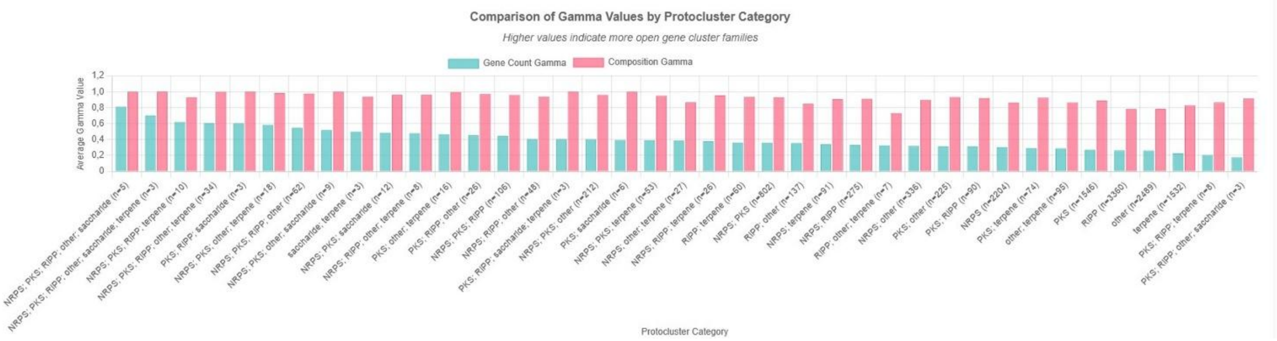

Supplementary Figure 3: Comparison of openness metrics across biosynthetic categories. Gene-based openness (Gene Count Gamma, teal bars) and composition-based openness (Composition Gamma, pink bars) for GCFs with ≥3 BGCs, stratified by biosynthetic class. Numbers in parentheses indicate the number of GCFs per category. Across all major biosynthetic classes and most hybrid combinations, composition-based openness consistently exceeds gene-based openness. Complex hybrids combining three or more biosynthetic classes show greater variability openness to new genes, though this may reflect small sample sizes for these rare cluster architectures. Higher gamma values indicate more open gene cluster families (ongoing expansion of diversity), while lower values indicate closed families (saturated diversity).
