# Supplementary material for: PanBGC: a pangenome-inspired framework for comparative analysis of biosynthetic gene clusters
Source: ISME Commun. 2025 Nov 27;5(1):ycaf225. doi: 10.1093/ismeco/ycaf225 (PMC12704434; doi:10.1093/ismeco/ycaf225)
Supplement: Supplementary_info_fig4_ycaf225 [file supplementary_info_fig4_ycaf225.pdf]

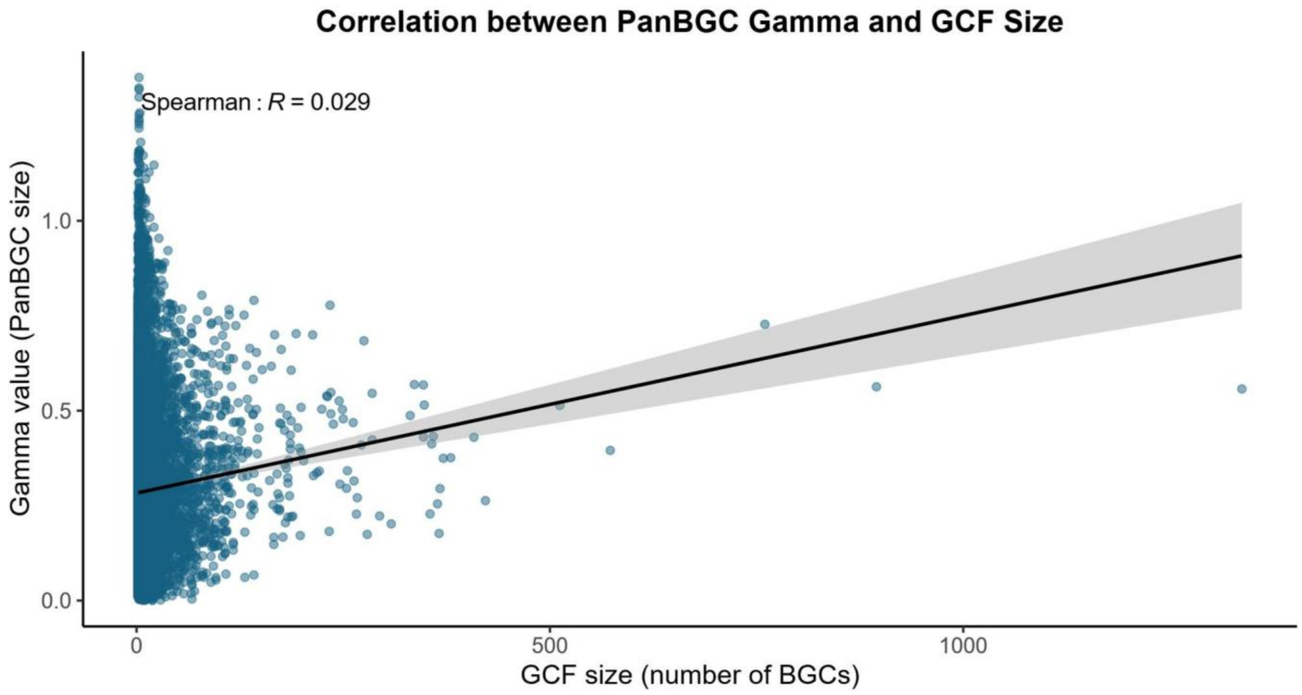

Supplementary Figure 4: Correlation between GCF size and PanBGC openness (γ-value). Scatterplot showing the relationship between the number of BGCs per gene cluster family (GCF size) and the corresponding gamma (γ) value calculated by the PanBGC framework, which quantifies openness based on Heaps' law. Each point represents a GCF. A slight positive trend is observed (Spearman's ρ = 0.029), indicating minimal correlation between family size and openness. The black line represents a linear regression fit with a 95% confidence interval (shaded area).
