# Supplementary material for: PanBGC: a pangenome-inspired framework for comparative analysis of biosynthetic gene clusters
Source: ISME Commun. 2025 Nov 27;5(1):ycaf225. doi: 10.1093/ismeco/ycaf225 (PMC12704434; doi:10.1093/ismeco/ycaf225)
Supplement: Supplementary_info_Tab1_ycaf225 [file supplementary_info_tab1_ycaf225.pdf]

TABLES

Supplementary Table 1: Overview of json, excel and nexus files used for data storage.

File	Description
**Overview.json**	Contains summary information about all GCFs. Used for overview table creation.
**mibig_compound.json**	Contains information about mibig compounds and their family. Used for Compound overview table.
**BGC_analysis_results.xlsx**	Contains statistics of pfam domains found in each class.
**Gamma_value_bgc_data.json**	Contains summary information about the gamma calculation of each GCF.
**gbk_inf.json**	Available for each GCF. Contains information about each cluster in the GCF, and stores domain structure of each BGC
**genbank_data.json**	Available for each GCF. Contains different annotations for each gene and cluster part of the GCF
**Heaps_law.json**	Available for some GCF. Contains simulation order for heap's law calculation
**Nexus.nex**	Available for some GCF. Stores OG trees and the coalescent tree in nexus format. Used for tanglegram creation.
**Report.json**	Available for each GCF. Contains summary of ZOL run for the GCF.

The files can be found under: https://github.com/ZiemertLab/PanBGC-DB/tree/master/Website_code/public/data
